# Supplementary material for: MICOS assembly controls mitochondrial inner membrane remodeling and crista junction redistribution to mediate cristae formation
Source: EMBO J. 2020 Jun 22;39(14):e104105. doi: 10.15252/embj.2019104105 (PMC7361284; doi:10.15252/embj.2019104105)
Supplement: Supplementary file 1 — Appendix [file EMBJ-39-e104105-s001.pdf]

Appendix to

MICOS assembly controls mitochondrial inner membrane remodeling and crista junction redistribution to mediate cristae formation

Till Stephan[a], Christian Brüser[a], Markus Deckers, Anna M. Steyer, Francisco Balzarotti, Mariam Barbot, Tiana S. Behr, Gudrun Heim, Wolfgang Hübner, Peter Ilgen, Felix Lange, David Pacheu-Grau, Jasmin Pape, Stefan Stoldt, Thomas Huser, Stefan W. Hell, Wiebke Möbius, Peter Rehling, Dietmar Riedel and Stefan Jakobs[b]

[a] Equal contributions

[b] To whom correspondence may be addressed. Email: sjakobs@gwdg.de

This Appendix includes:

Appendix Figures S1 – S6

Appendix Table S1

Appendix Figure S1

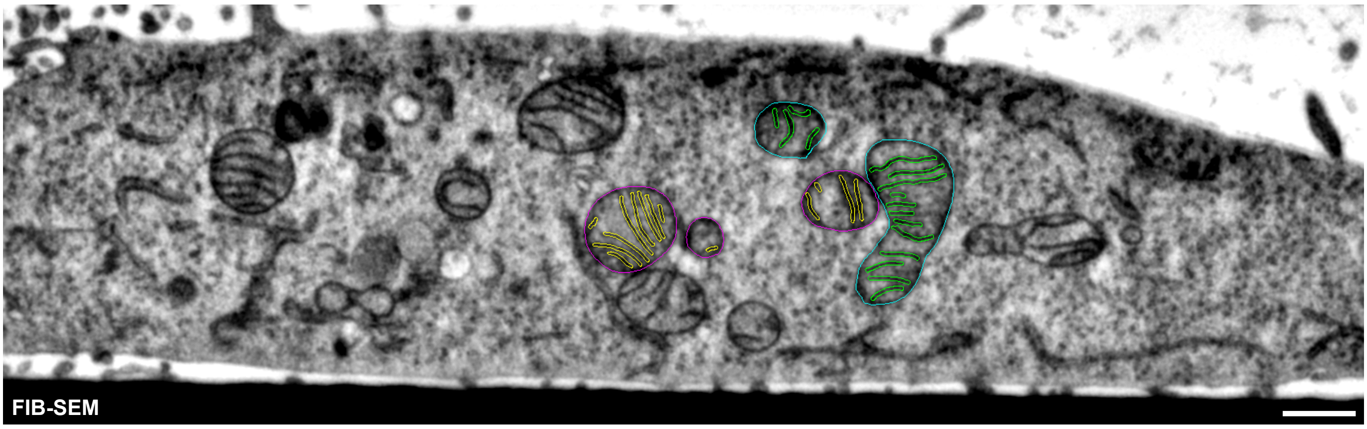

Appendix Figure S1. Inner membrane architecture in HeLa cells.

(A) FIB-SEM of HeLa WT cells. Shown is one section of a FIB-SEM stack. Reconstructed mitochondria (colored lines) are shown in Fig 1B. Scale bar: 1 μm.

Appendix Figure S2

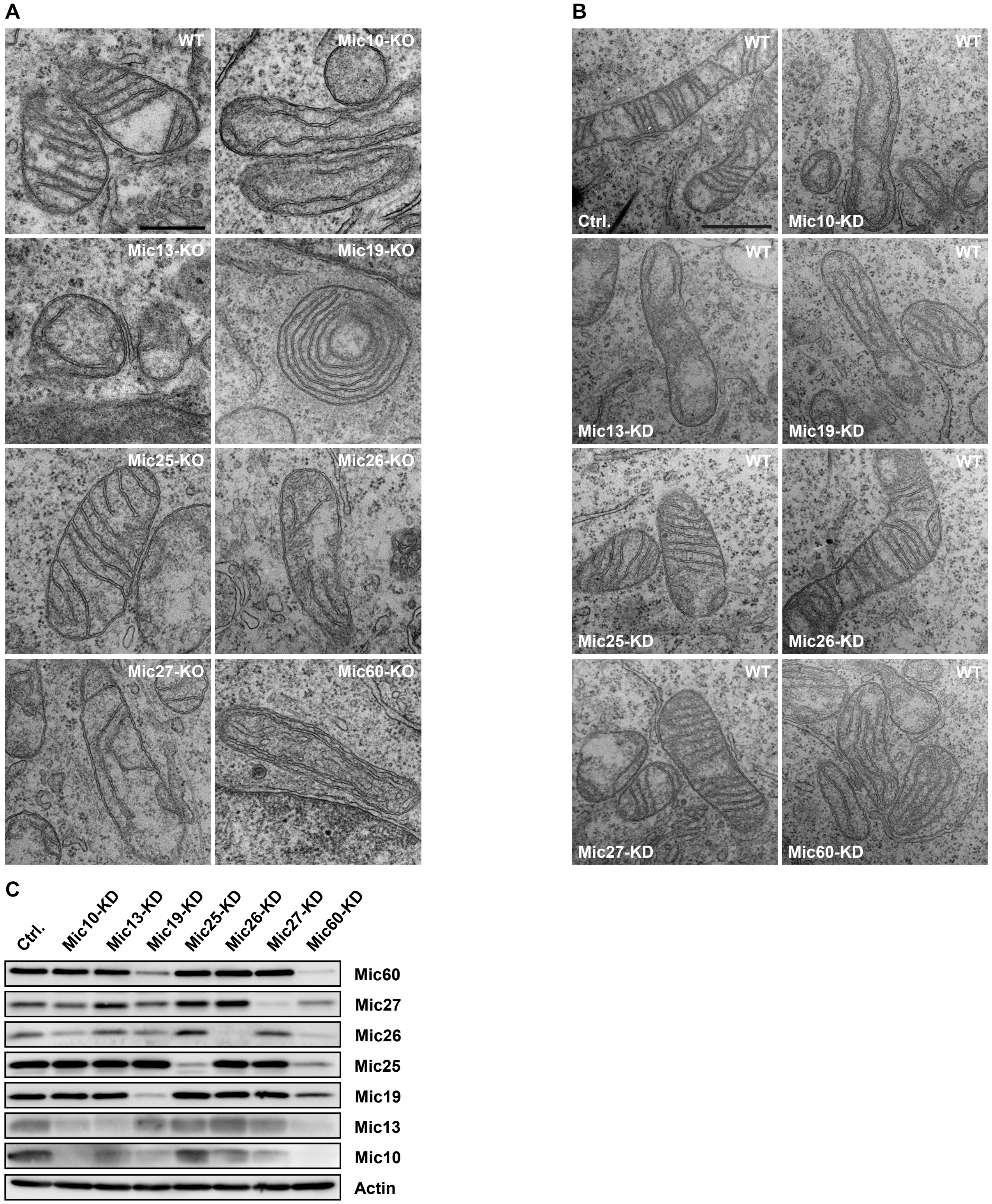

Appendix Figure S2. Depletion of MICOS subunits affects the formation of lamellar cristae. (A-B) Comparison of MICOS knockouts (KO) with MICOS knockdowns (KD). (A) Representative TEM recordings of the KO-cell lines indicated. (B,C) Knockdown of MICOS subunits using siRNA. WT cells were transfected with a scrambled control (Ctrl.) or specific siRNA pools and analyzed after 48h. (B) Images show representative TEM recordings. (C) Protein levels of MICOS subunits in cell lysates analyzed by western blotting. Scale bars: 500 nm (A), 1 µm (B).

Appendix Figure S3

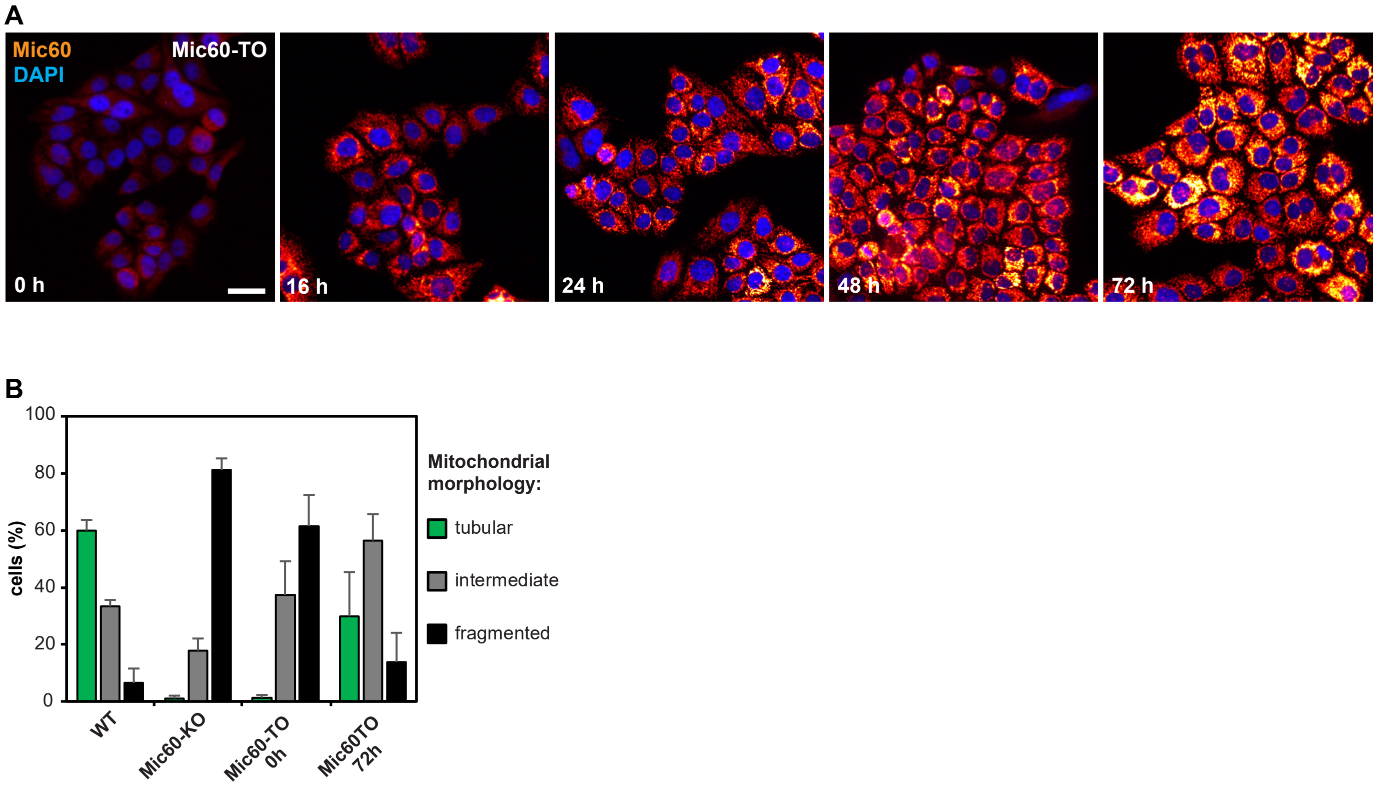

Appendix Figure S3. Re-expression of Mic60 in Mic60-TO cells rescues the morphology of the mitochondrial network. (A-B) Rescue of Mic60 expression in Mic60-TO cells upon induction with doxycycline. (A) Cells were immunolabeled for Mic60 and DNA and visualized by confocal fluorescence microscopy at the indicated time points after induction of Mic60 expression. (B) Quantification of mitochondrial network remodeling upon Mic60 re-expression. For the analysis, cells were immunolabeled for TOM20, imaged by confocal fluorescence microscopy, and manually annotated. Average and SD of three independent biological repeats are shown. For each repeat, at least 170 cells per sample were analyzed. Scale bar: 40 µm.

Appendix Figure S4

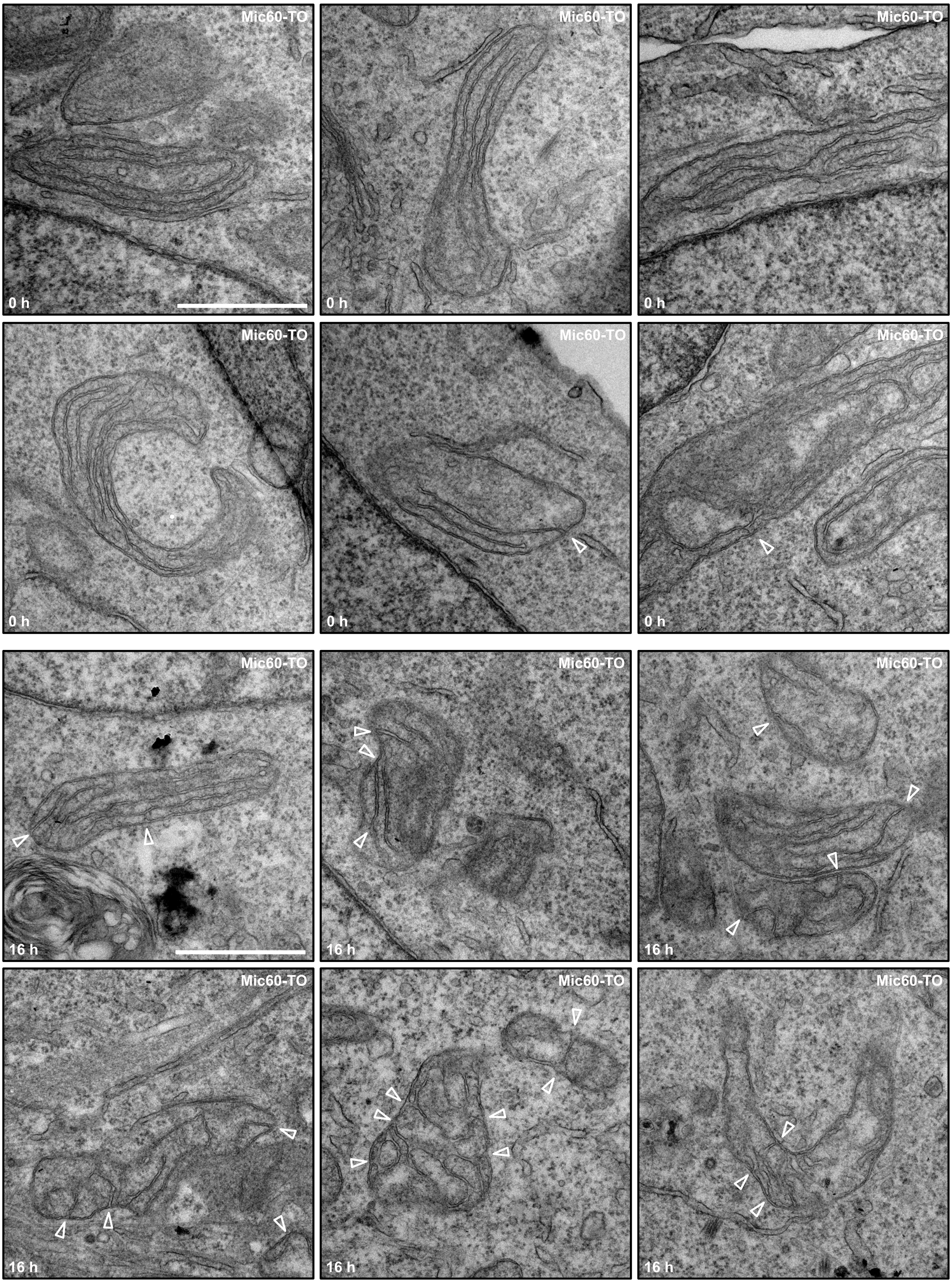

Appendix Figure S4. Mic60 controls the formation of secondary CJs. TEM recordings of Mic60-TO cells induced with doxycycline for 0 h and 16 h to facilitate the expression of Mic60. Arrows mark CJs. Scale bars: 1 µm.

Appendix Figure S5

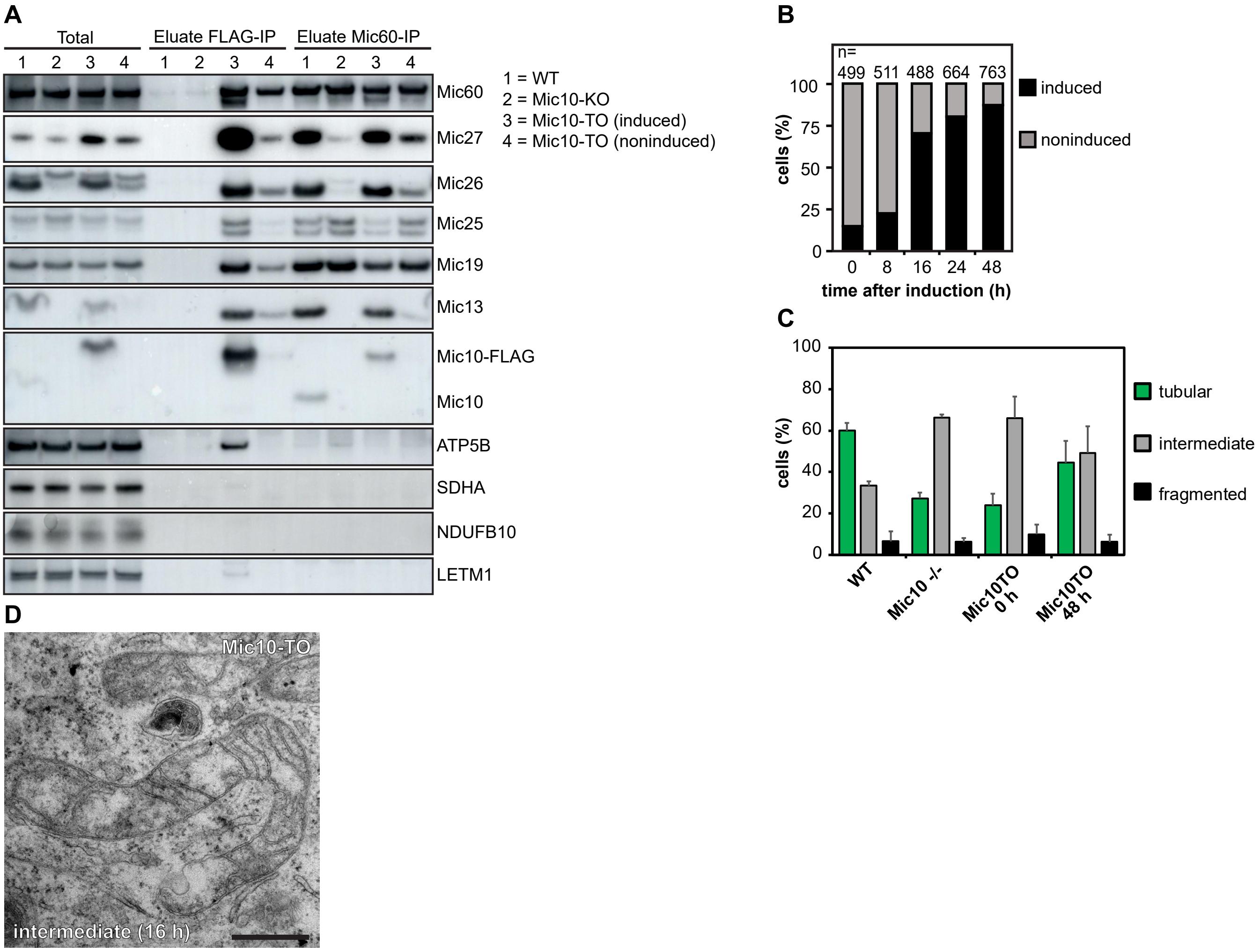

Appendix Figure S5. Re-expression of Mic10-FLAG in Mic10-TO cells induces the formation of the MICOS holo-complex and rescues the morphology of the mitochondrial network. (A) MICOS-subunit expression levels in WT, Mic10-KO and induced or noninduced Mic10-TO cells. FLAG antibodies were used to pull-down Mic10-FLAG and interacting proteins (eluate) from cell lysates (total). Mic60-antibodies were used to pull-down Mic60 and interacting proteins from cell lysates. Samples were analyzed by western blotting. (B) Quantification of induced Mic10-TO cells. Cells were induced with doxycycline for the indicated time periods and analyzed. Cells that expressed visible amounts of Mic10 were counted as induced. n: number of cells analyzed. (C) Quantification of mitochondrial network remodeling upon Mic10-FLAG re-expression in Mic10-TO cells. For the analysis, cells were immunolabeled for TOM20, imaged by confocal fluorescence microscopy, and manually annotated. Average and SD of three independent biological repeats are shown. For each repeat, at least 170 cells per sample were analyzed. (D) Remodeling of the CMs. Induced Mic10-TO cells were analyzed by TEM. The image shows a representative mitochondrion with an intermediate cristae architecture after 16 h of induction. Scale bar: 1 µm.

Appendix Figure S6

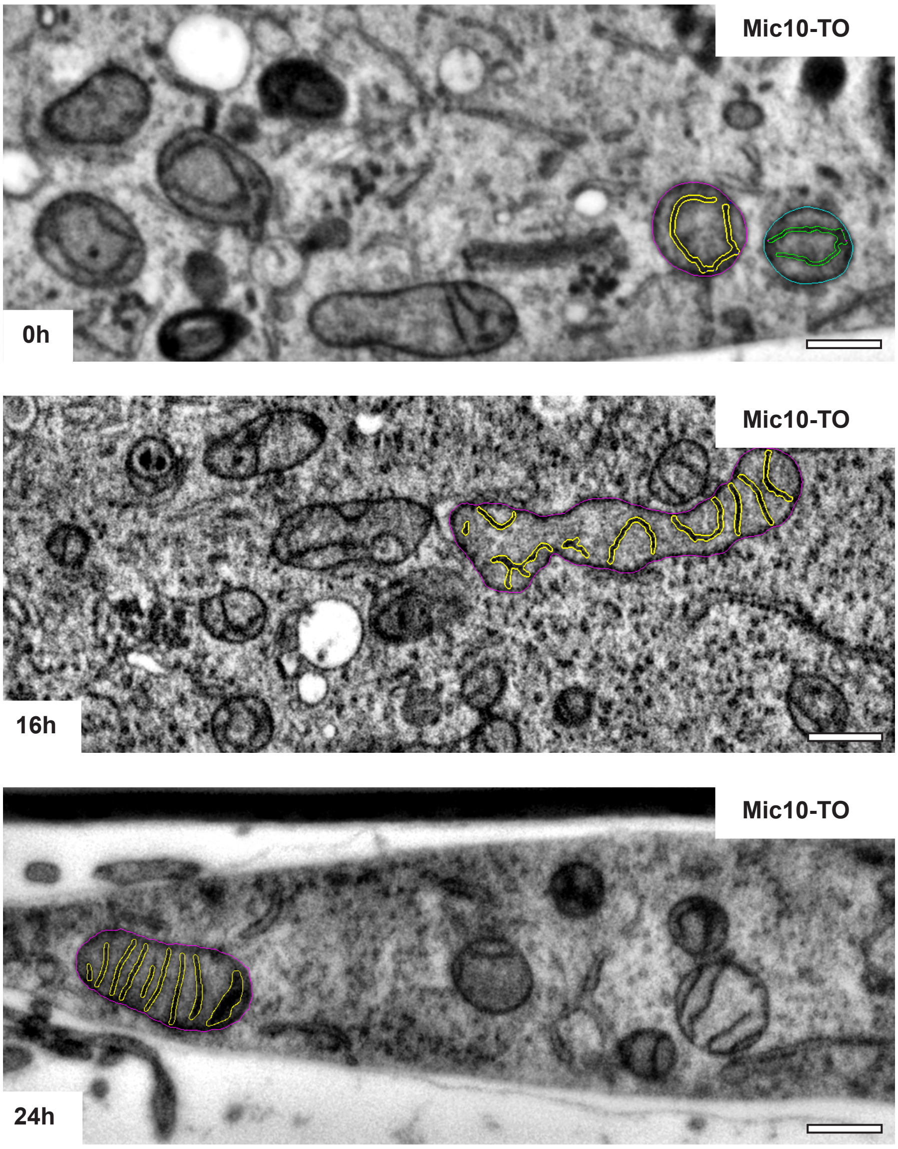

Appendix Figure S6. MICOS holo-complex assembly induces remodeling of the inner membrane. FIB-SEM of Mic10-TO cells after induction of re-expression of Mic10-FLAG. Cells were induced for 0 h, 16 h and 24 h and analyzed with FIB-SEM. Shown are single sections of representative FIB-SEM stacks. The mitochondria that were reconstructed for Fig 7A are highlighted by colored lines. Scale bars: 1 μm.

Appendix Table S1

MICOS Subunit	Target Exon	Allele	Mutation	Sequence (5'-3')	Frequency
**Mic10**	Exon1	Wt		CGGAGCTCGGCAGGA	0%
		Mut1	Nonsense: Insertion leading to a frameshift and an early stop codon in the first exon	CGGAGCTC**TC**GGCAGGA	75%
		Mut2	Nonsense: Deletion leading to a frameshift and an early stop codon in the first exon	CGGAGCT - GGCAGGA	25%
**Mic13**	Exon 3	Wt		CGCCGTCTACCTGGT	0%
		Mut1	Nonsense: Insertion leading to a frameshift and an early stop codon in the fourth exon	CGCCGTCT**T**ACCTGGT	72%
		Mut2	Nonsense: Deletion leading to a frameshift and an early stop codon in the fourth exon	CGCCGTCT - - CTGGT	28%
**Mic19**	Exon1	Wt		GAACATCACCGTGGT	0%
		Mut1	Nonsense: Deletion leading to a frameshift and an early stop codon in the second exon	GAACATCA - CGTGGT	72%
		Mut2	Nonsense: Insertion leading to a frameshift and an early stop codon in the second exon	GAACATCA**A**CCGTGGT	28%
**Mic25**	Exon2	Wt		CTTTGGCCTTCAAGA	0%
		Mut1	Nonsense: Deletion leading to a frameshift and an early stop codon in the second exon	CTTTGGCC - - - - AGA	68%
		Mut2	Nonsense: Deletion leading to a frameshift and an early stop codon in the fourth exon	CTTTGGCCT - - AAGA	32%
**Mic26**	Exon4	Wt		TCAGTTCCTGAGGGT	0%
		Mut1	Nonsense: Insertion leading to a frameshift and an early stop codon in the fourth exon	TCAGTTCC**C**TGAGGGT	79%
		Mut2	Nonsense: Deletion leading to a frameshift and an early stop codon in the fifth exon	TCAGTTCC - GAGGGT	21%
**Mic27**	Exon3	Wt		GGTTGTTACATTGGC	0%
		Mut1	Nonsense: Deletion leading to a frameshift and an early stop codon in the fifth exon	GGTTGTTA - - TTGGC	100%
**Mic60**	Exon1	wt		GGGCCTGTCAGTTAT	0%
		Mut1	Nonsense: Deletion leading to a frameshift and an early stop codon in the first exon	GGGCCTGT - - - - TAT	55%
		Mut2	Nonsense: A Part of the first Exon is missing, together with a huge insertion leading to an early stop codon in the first exon	- - - - - - - - - - - - - - -	45%

Appendix Table S1. CRISPR/Cas9 mediated disruption of MICOS genes in HeLa cells.
For the generation of KO-cell lines, gRNAs against the shown target sequences were used. Cell lines were verified by sequencing as well as western blotting. For sequencing, PCR products were sub-cloned. Approximately 20 sub-clones were sequenced for each cell line. The table indicates the frequency of specific indels found by sequencing of the sub-clones for each cell line. No WT alleles could be identified for any of the MICOS mutant cell lines.
