# Supplementary material for: MICOS assembly controls mitochondrial inner membrane remodeling and crista junction redistribution to mediate cristae formation
Source: EMBO J. 2020 Jun 22;39(14):e104105. doi: 10.15252/embj.2019104105 (PMC7361284; doi:10.15252/embj.2019104105)
Supplement: Supplementary file 2 — Expanded View Figures PDF [file EMBJ-39-e104105-s002.pdf]

Expanded View Figures

Figure EV1. MICOS distribution in HeLa cells and characterization of MICOS knockout cell lines.

A Left: STED nanoscopy of mitochondria immunolabeled for Mic60 and dsDNA. A part of the image is also shown in Fig 1D. Right: Line profiles of the fluorescence intensities were measured at six sites exhibiting mtDNA (blue arrows) and at six sites devoid of mtDNA (white arrows), as indicated. The six line profiles of each condition were normalized and averaged, respectively.

B Dual-color STED nanoscopy of cells labeled for COX8A-SNAP and immunolabeled for Mic60. A part of the image is also shown in Fig 1E.

C Protein levels of mitochondrial proteins in cell lysates from WT cells and MICOS mutants. Cell lysates from the indicated cell lines were analyzed by Western blotting.

D BN–PAGE of isolated mitochondria. Indicated cell lines were analyzed by BN–PAGE and Western blotting.

Data information: Scale bars: 1 μm.

Source data are available online for this figure.

© 2020 The Authors

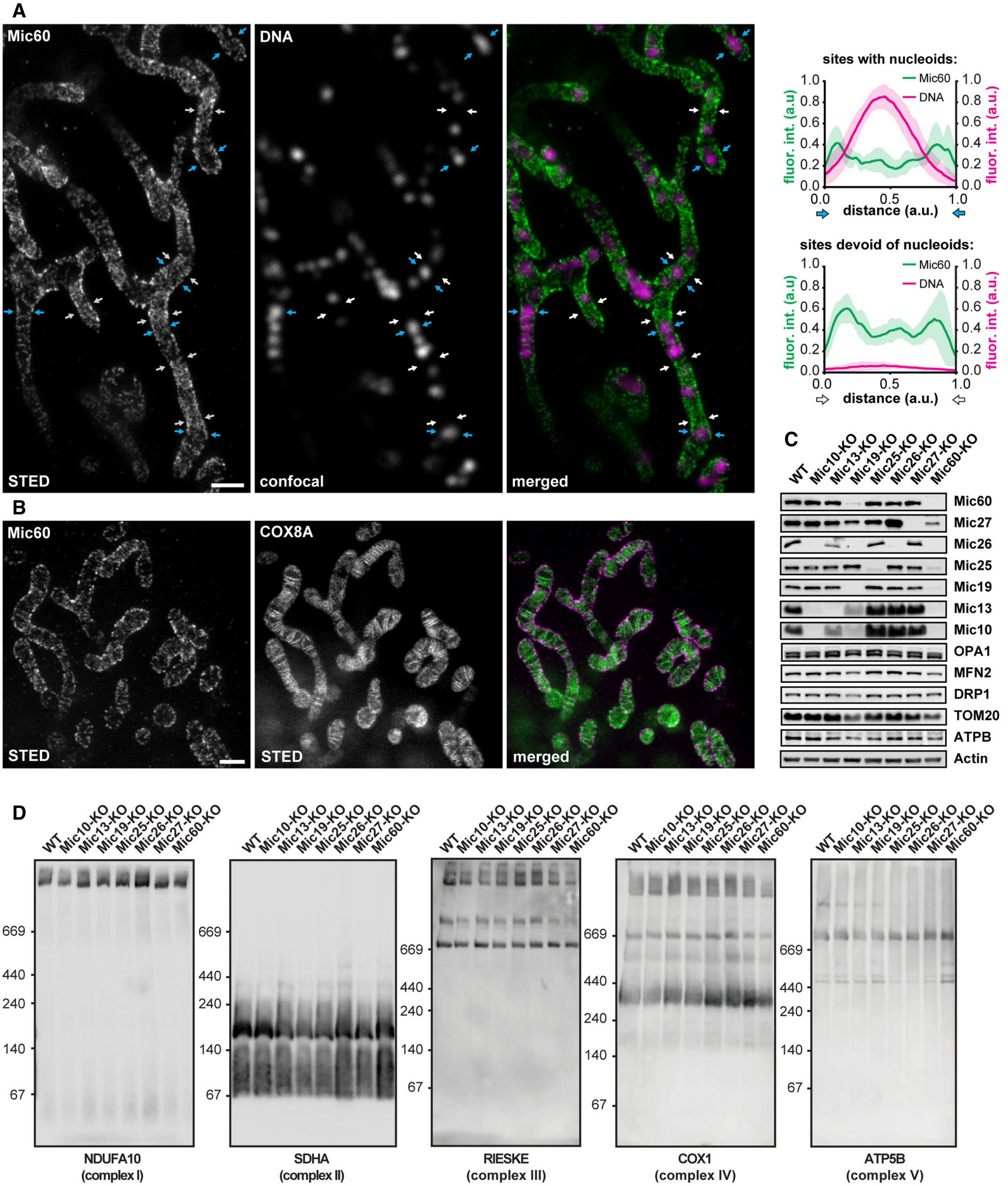

Figure EV1.

© 2020 The Authors

Figure EV2. Depletion of Mic10 causes the formation of large tube-like cristae and opposite distribution bands of Mic60, Mic19, and ATPB.

A–C Nanoscopy of mitochondria. (A) WT and Mic10-KO cells were immunolabeled for Mic19 and ATPB and visualized by 2D STED nanoscopy. (B) WT and Mic13-KO cells were immunolabeled for Mic60 and ATPB and recorded with 2D STED nanoscopy. (C) 3D MINFLUX nanoscopy of mitochondria. WT cells were immunolabeled for Mic60. Colors encode depth information.

D 3D SIM of live cells. Mic10-KO cells were labeled with Mitotracker Green. Images show a cross section (side view) through several mitochondria in a 3D SIM stack.

E STED nanoscopy of mitochondria. WT and Mic19-KO cells were immunolabeled for Mic60 and ATPB and analyzed by STED nanoscopy.

F Nanoscopy of mitochondria. Mic19-KO cells were transfected with a plasmid for Mic10-FLAG expression under the control of a tetracycline promoter and induced with doxycycline for 24 h. Inset 1: mitochondria from a cell strongly expressing Mic10-FLAG. Inset 2: mitochondria from a cell expressing virtually no Mic10-FLAG.

Data information: Scale bars: 1 µm (A–E, and F, lower panel), 10 µm (F, upper panel).

© 2020 The Authors

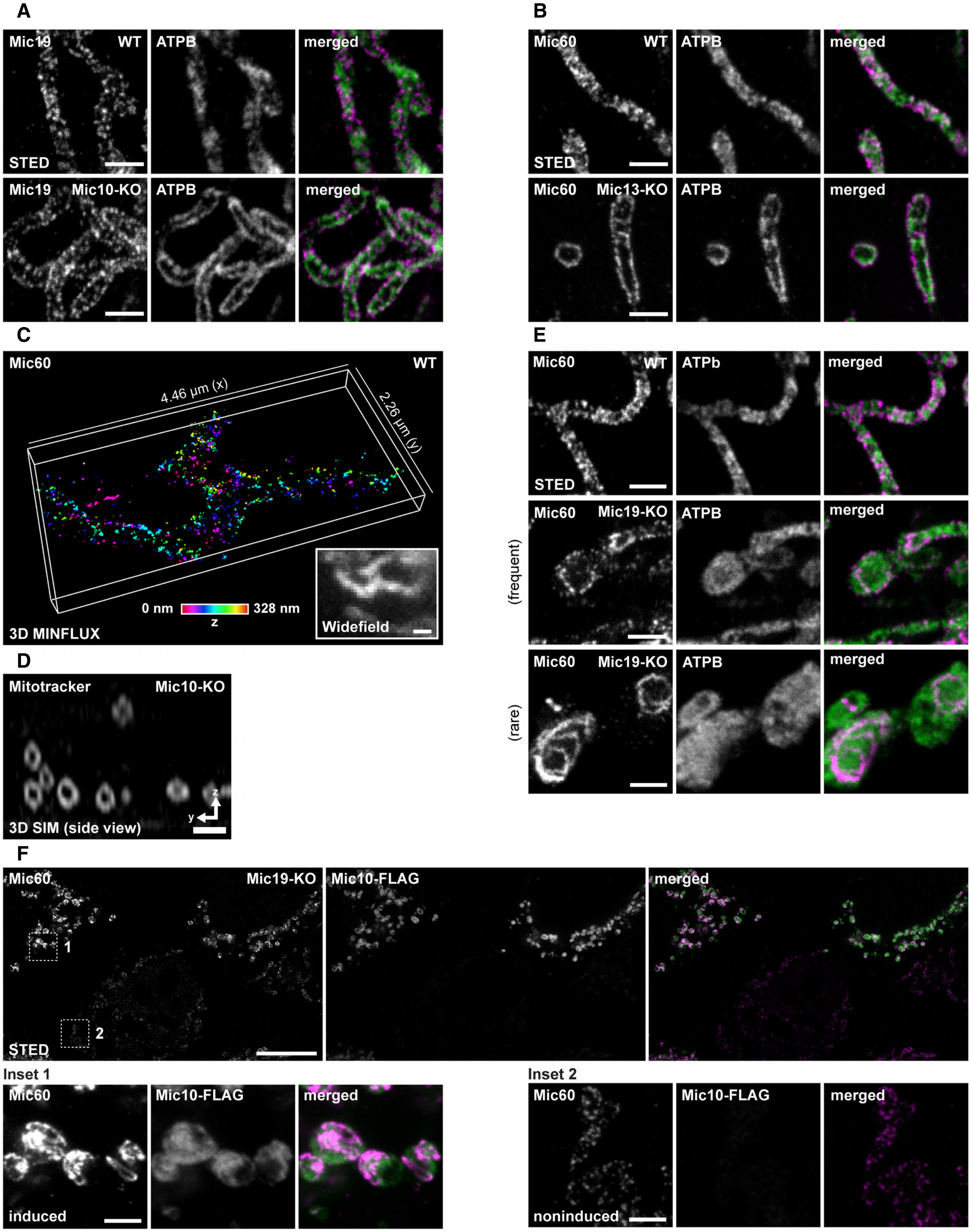

Figure EV2.

© 2020 The Authors

Figure EV3. The Mic10-subcomplex is essential for coordination of lamellar cristae formation.

A–C Expression of Mic10-FLAG upon induction of Mic10-TO cells. (A) Cells were induced with doxycycline hyclate, immunolabeled for Mic10-FLAG, stained with DAPI, and subsequently recorded by confocal fluorescence microscopy. (B) Composition of MICOS in induced Mic10-TO cells. FLAG antibodies were used to pull down Mic10-FLAG and interacting proteins from cell lysates. Mic60-antibodies were used to pull down Mic60 and interacting proteins from cell lysates. Samples were analyzed by Western blotting. (C) Nanoscopy of Mic10-TO cells. Cells were immunolabeled for Mic60. Images show 2D STED recordings of cells induced for the indicated time period. A dual-color cutout of the same image (24 h) is shown in Fig 6C.

D, E Dual-color STED nanoscopy of Mic10-TO cells. Cells were induced for the indicated time periods and immunolabeled for Mic60 and Mic10-FLAG or ATPB and Mic10-FLAG, respectively. A larger single-color overview including the same image for 0 h induction is shown in (C).

Data information: Scale bars: 20 µm (A), 2 µm (C), 1 µm (D, E).

Source data are available online for this figure.

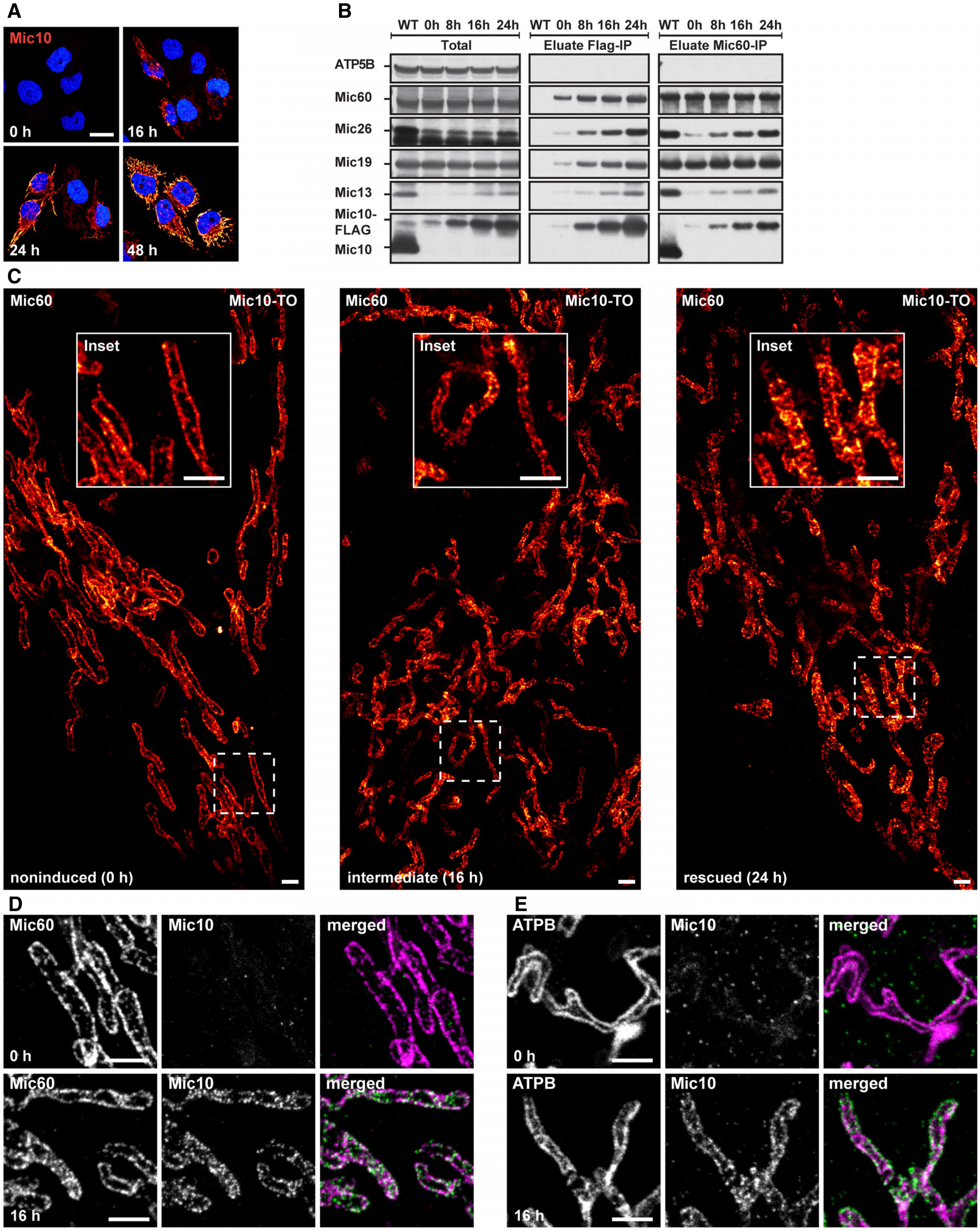

Figure EV3.

 © 2020 The Authors

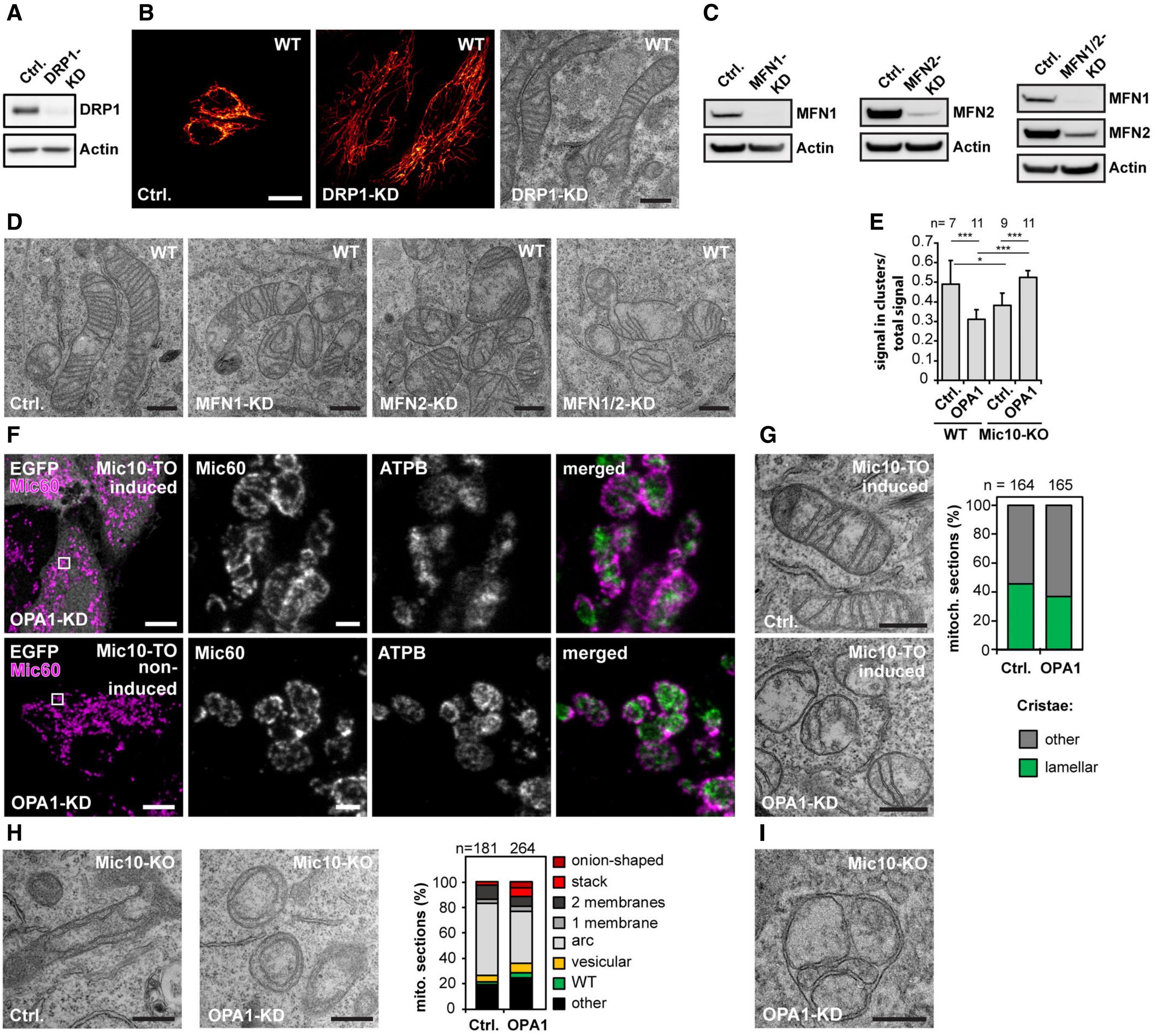
A
Ctrl.
DRP1-KD
DRP1
Actin
B
WT
Ctrl.
DRP1-KD
C
MFN1-KD
MFN2-KD
MFN1/2-KD
MFN1
MFN2
D
MFN1-KD
MFN2-KD
MFN1/2-KD
E
n= 7 11 9 11
signal in clusters/ total signal
OPA1
WT
Mic10-KO
F
EGFP
Mic60
Mic10-TO induced
Mic10-TO non-induced
OPA1-KD
ATPB
merged
G
n = 164 165
mitoch. sections (%)
Cristae:
other
lamellar
H
n=181 264
mito. sections (%)
onion-shaped
stack
2 membranes
1 membrane
arc
vesicular
I

Figure EV4.

Figure EV4. Formation of lamellar cristae does not require mitochondrial tubule fusion or fission; Mic10 influences the distribution of Mic60.

A, B Knockdown (KD) of DRP1 in HeLa cells. WT cells were transfected with a scrambled control (Ctrl.) or a siRNA plasmid against DRP1 for 7 days. (A) Cells were analyzed by Western blotting. (B) Ctrl. and DRP1-KD cells were immunolabeled for TOM20 and analyzed by confocal fluorescence microscopy (left) or analyzed by TEM (right).

C, D KD of MFN1 and MFN2 in HeLa cells. WT cells were transfected with Ctrl. or siRNA pools against MFN1, MFN2, or both proteins together for 4 days. (C) Cells were analyzed by Western blotting. (D) Cells were analyzed by TEM.

E Mic60 cluster analysis of OPA1-deficient HeLa cells. WT cells and Mic10-KO cells were either transfected with a scrambled control (Ctrl.) or a siRNA pool against OPA1(OPA1-KD). Cells were immunolabeled against Mic60 and recorded by 2D STED nanoscopy. Images were automatically segmented and the ratio of Mic60 signals in small distinct clusters (Mic60 assemblies $\leq 0.00225\ \mu m^2$) to the total Mic60 signals was determined. Higher values indicate smaller Mic60 assemblies and lower values the formation of larger Mic60 assemblies. Bars indicate mean. Error bars indicate SD. For statistics, a one-way ANOVA assay was performed. *$P \leq 0.05$, ***$P \leq 0.001$. *n*: Number of analyzed cells.

F, G Mic10 re-expression after OPA1 depletion. Mic10-TO cells were transfected with a scrambled control (Ctrl.) or a siRNA pool against OPA1 (OPA1-KD) for 48 h. Afterward, Mic10-FLAG expression was induced for 24 h. (F) Cells were immunolabeled for Mic60 and ATPB and recorded by STED nanoscopy. Left panel: Confocal overview images taken from induced cells (cytosolic reporter EGFP, upper panel) and noninduced cells (lower panel). Right: STED images from mitochondria from the areas indicated by rectangles. (G) TEM of Ctrl. and OPA1-deficient Mic10-TO cells after re-expression of Mic10-FLAG. Right: quantification of the cristae morphology. *n*: Number of mitochondrial sections analyzed.

H, I Knockdown of OPA1 in Mic10-KO cells. (H) Left: Cells were transfected with a Ctrl. or an siRNA pool against OPA1 and analyzed by TEM. Right: Recordings were assigned to the indicated crista phenotypes. *n*: Number or mitochondrial sections analyzed. (I) Recording of a mitochondrion of a MIC10-KO cell depleted of OPA1 exhibiting septa and septa junctions.

Data information: Scale bars: 20 μm (B, light microscopy), 10 μm (F, overview), 500 nm (B, TEM; D; F, inset; G; H), 250 nm (I).

Source data are available online for this figure.

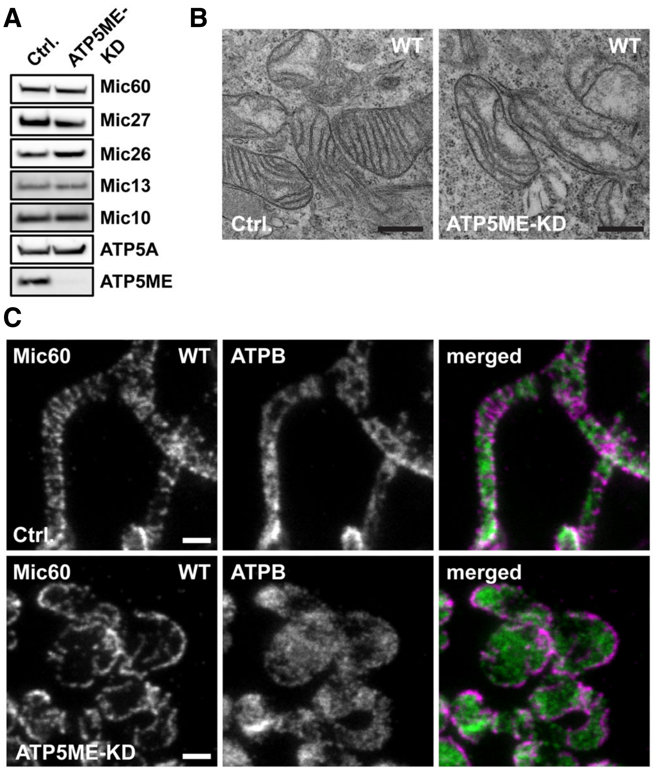

Figure EV5. Dimers of the F_1F_o-ATP synthase influence the MICOS distribution.

Knockdown of ATP5ME in HeLa cells. Cells were transfected with a Ctrl. or an siRNA pool against ATP5ME (ATP5ME-KD) and analyzed after 4 days.

A Protein levels of ATP5ME, ATP5A, and MICOS proteins in cell lysates as analyzed by Western blotting.

B TEM recordings of ATP5ME-depleted cells.

C STED nanoscopy of ATP5ME-depleted cells. Cells were immunolabeled for Mic60 and ATPB.

Data information: Scale bars: 500 nm.

Source data are available online for this figure.

 © 2020 The Authors
