# Supplementary material for: MICOS assembly controls mitochondrial inner membrane remodeling and crista junction redistribution to mediate cristae formation
Source: EMBO J. 2020 Jun 22;39(14):e104105. doi: 10.15252/embj.2019104105 (PMC7361284; doi:10.15252/embj.2019104105)
Supplement: Supplementary file 3 — Movie EV1 [file EMBJ-39-e104105-s003.zip › Movie EV1.docx]

**Movie EV1. Cristae architecture of mitochondria from HeLa WT cells.** Two mitochondria were reconstructed from a FIB-SEM stack. The cristae are shown in blue, the OM and IBM are shown together in clear grey. A twisted crista is highlighted in grey. A still image is shown in Fig 1B.
