# Supplementary material for: MICOS assembly controls mitochondrial inner membrane remodeling and crista junction redistribution to mediate cristae formation
Source: EMBO J. 2020 Jun 22;39(14):e104105. doi: 10.15252/embj.2019104105 (PMC7361284; doi:10.15252/embj.2019104105)
Supplement: Supplementary file 4 — Movie EV2 [file EMBJ-39-e104105-s004.zip › Movie EV2.docx]

**Movie EV2. Live-cell STED nanoscopy of Mic10-SNAP.** Mic10-SNAP was expressed in HeLa cells and labeled with SNAP-cell SiR. Mitochondria were recorded with STED nanoscopy every 15 seconds. Photobleaching was compensated.
