# Supplementary material for: MICOS assembly controls mitochondrial inner membrane remodeling and crista junction redistribution to mediate cristae formation
Source: EMBO J. 2020 Jun 22;39(14):e104105. doi: 10.15252/embj.2019104105 (PMC7361284; doi:10.15252/embj.2019104105)
Supplement: Supplementary file 5 — Movie EV3 [file EMBJ-39-e104105-s005.zip › Movie EV3.docx]

**Movie EV3. ET of HeLa Mic60-KO cells.** A mitochondrion was reconstructed from a tilt series recorded by TEM. The OM is displayed in clear grey, the side of the IM that faces the matrix is shown in dark blue. The IM side that faces the inter membrane space is shown in light blue. A still image in shown in Fig 3F.
