# Supplementary material for: MICOS assembly controls mitochondrial inner membrane remodeling and crista junction redistribution to mediate cristae formation
Source: EMBO J. 2020 Jun 22;39(14):e104105. doi: 10.15252/embj.2019104105 (PMC7361284; doi:10.15252/embj.2019104105)
Supplement: Supplementary file 6 — Movie EV4 [file EMBJ-39-e104105-s006.zip › Movie EV4.docx]

**Movie EV4. ET of HeLa Mic60-KO cells.** Tilt series of mitochondria recorded by TEM.
