# Supplementary material for: MICOS assembly controls mitochondrial inner membrane remodeling and crista junction redistribution to mediate cristae formation
Source: EMBO J. 2020 Jun 22;39(14):e104105. doi: 10.15252/embj.2019104105 (PMC7361284; doi:10.15252/embj.2019104105)
Supplement: Supplementary file 9 — Movie EV7 [file EMBJ-39-e104105-s009.zip › Movie EV7.docx]

**Movie EV7. ET of HeLa Mic10-KO cells.** Tilt series of a mitochondrion with an onion-shaped cristae architecture recorded by TEM.
