# Supplementary material for: MICOS assembly controls mitochondrial inner membrane remodeling and crista junction redistribution to mediate cristae formation
Source: EMBO J. 2020 Jun 22;39(14):e104105. doi: 10.15252/embj.2019104105 (PMC7361284; doi:10.15252/embj.2019104105)
Supplement: Supplementary file 10 — Movie EV8 [file EMBJ-39-e104105-s010.zip › Movie EV8.docx]

**Movie EV 8. Live-cell STED nanoscopy of mitochondria from Mic10-KO cells.** Cells expressing COX8A-SNAP were stained with SNAP-cell SiR and visualized by time-lapse STED nanoscopy every 5 seconds.
