# Supplementary material for: MICOS assembly controls mitochondrial inner membrane remodeling and crista junction redistribution to mediate cristae formation
Source: EMBO J. 2020 Jun 22;39(14):e104105. doi: 10.15252/embj.2019104105 (PMC7361284; doi:10.15252/embj.2019104105)
Supplement: Supplementary file 11 — Movie EV9 [file EMBJ-39-e104105-s011.zip › Movie EV9.docx]

**Movie EV9. Live-cell STED nanoscopy of mitochondria from Mic10-KO cells.** Cells expressing COX8A-SNAP were stained with SNAP-cell SiR and visualized by time-lapse STED nanoscopy every 15 seconds.
