# Supplementary material for: MICOS assembly controls mitochondrial inner membrane remodeling and crista junction redistribution to mediate cristae formation
Source: EMBO J. 2020 Jun 22;39(14):e104105. doi: 10.15252/embj.2019104105 (PMC7361284; doi:10.15252/embj.2019104105)
Supplement: Supplementary file 12 — Movie EV10 [file EMBJ-39-e104105-s012.zip › Movie EV10.docx]

**Movie EV10.** **FIB-SEM of Mic10-TO cells.** Cristae architecture of mitochondria from a noninduced Mic10-TO cell. A mitochondrion was reconstructed from a FIB-SEM stack. The CM is shown in blue, the OM and IBM are shown together in clear grey. A still image is shown in Fig 7A.
