# Supplementary material for: MICOS assembly controls mitochondrial inner membrane remodeling and crista junction redistribution to mediate cristae formation
Source: EMBO J. 2020 Jun 22;39(14):e104105. doi: 10.15252/embj.2019104105 (PMC7361284; doi:10.15252/embj.2019104105)
Supplement: Supplementary file 14 — Movie EV12 [file EMBJ-39-e104105-s014.zip › Movie EV12.docx]

**Movie EV12. FIB-SEM of Mic10-TO cells.** Cristae architecture of mitochondria from a Mic10-TO cell induced for Mic10 re-expression for 24 h. A mitochondrion was reconstructed from a FIB-SEM stack. The CM is shown in blue, the OM and IBM are shown together in clear grey. A still image is shown in Fig 7A.
