# Supplementary material for: MICOS assembly controls mitochondrial inner membrane remodeling and crista junction redistribution to mediate cristae formation
Source: EMBO J. 2020 Jun 22;39(14):e104105. doi: 10.15252/embj.2019104105 (PMC7361284; doi:10.15252/embj.2019104105)
Supplement: Supplementary file 15 — Movie EV13 [file EMBJ-39-e104105-s015.zip › Movie EV13.docx]

**Movie EV13. ET of Mic10-TO cells.** A tilt series was recorded from a noninduced cell. Mitochondria were reconstructed. The OM is displayed in clear grey, the side of the IM that faces the matrix is shown in dark blue. The IM side that faces the inter membrane space is shown in light blue. A still image is shown in Fig 7B.
