# Supplementary material for: MICOS assembly controls mitochondrial inner membrane remodeling and crista junction redistribution to mediate cristae formation
Source: EMBO J. 2020 Jun 22;39(14):e104105. doi: 10.15252/embj.2019104105 (PMC7361284; doi:10.15252/embj.2019104105)
Supplement: Supplementary file 16 — Movie EV14 [file EMBJ-39-e104105-s016.zip › Movie EV14.docx]

**Movie EV14. ET of Mic10-TO cells.** A tilt series was recorded from a cell induced for Mic10 re-expression for 16 h. Mitochondria were reconstructed. The OM is displayed in clear grey, the side of the IM that faces the matrix is shown in dark blue. The IM side that faces the inter membrane space is shown in light blue. A still image is shown in Fig 7B.
