# Supplementary material for: MICOS assembly controls mitochondrial inner membrane remodeling and crista junction redistribution to mediate cristae formation
Source: EMBO J. 2020 Jun 22;39(14):e104105. doi: 10.15252/embj.2019104105 (PMC7361284; doi:10.15252/embj.2019104105)
Supplement: Supplementary file 17 — Movie EV15 [file EMBJ-39-e104105-s017.zip › Movie EV15.docx]

**Movie EV15. ET of HeLa control cells.** Cells were transfected with a scrambled control for 48 h. A tilt series was recorded and a mitochondrion was reconstructed. The OM is shown in grey, the IM in blue. The side of the IM that faces the inter membrane space is shown in light blue and the side that faces the matrix space is shown in dark blue. A still image is shown in Fig 8E.
