# Supplementary material for: MICOS assembly controls mitochondrial inner membrane remodeling and crista junction redistribution to mediate cristae formation
Source: EMBO J. 2020 Jun 22;39(14):e104105. doi: 10.15252/embj.2019104105 (PMC7361284; doi:10.15252/embj.2019104105)
Supplement: Supplementary file 18 — Movie EV16 [file EMBJ-39-e104105-s018.zip › Movie EV16.docx]

**Movie EV16. ET of HeLa OPA1-KD cells.** Cells were transfected with siRNA pools against OPA1 for 48 h. A tilt series was recorded and mitochondria were reconstructed. The OM is shown in grey, the IM in blue. The side of the OM that faces the inter membrane space is shown in light blue and the side that faces the matrix space is shown in dark blue. A still image is shown in Fig 8F.
