# Supplementary material for: MICOS assembly controls mitochondrial inner membrane remodeling and crista junction redistribution to mediate cristae formation
Source: EMBO J. 2020 Jun 22;39(14):e104105. doi: 10.15252/embj.2019104105 (PMC7361284; doi:10.15252/embj.2019104105)

Paper Title:	MICOS assembly controls mitochondrial inner membrane remodeling and crista junction redistribution to mediate cristae formation
First Author:	Till Stephan
Corresponding Author:	Stefan Jakobs
Authors:	Till Stephan, Christian Brüser, Markus Deckers, Anna M. Steyer, Francisco Balzarotti, Mariam Barbot, Tiana S. Behr, Gudrun Heim, Wolfgang Hübner, Peter Ilgen, Felix Lange, David Pacheu-Grau, Jasmin Pape, Stefan Stoldt, Thomas Huser, Stefan W. Hell, Wiebke Möbius, Peter Rehling, Dietmar Riedel, Stefan Jakobs
Figure:	EV5
Panel:	A
Page:	1 of 2

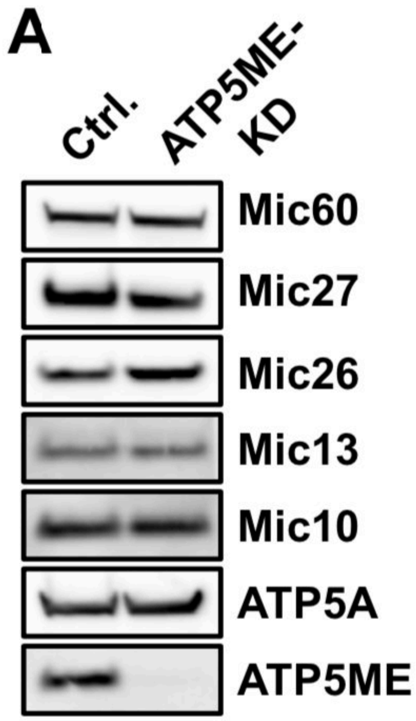

Paper Title: MICOS assembly controls mitochondrial inner membrane remodeling and crista junction redistribution to mediate cristae formation

First Author: Till Stephan

Corresponding Author: Stefan Jakobs

Authors: Till Stephan, Christian Brüser, Markus Deckers, Anna M. Steyer, Francisco Balzarotti, Mariam Barbot, Tiana S. Behr, Gudrun Heim, Wolfgang Hübner, Peter Ilgen, Felix Lange, David Pacheu-Grau, Jasmin Pape, Stefan Stoldt, Thomas Huser, Stefan W. Hell, Wiebke Möbius, Peter Rehling, Dietmar Riedel, Stefan Jakobs

Figure: EV5

Panel: A

Page: 2 of 2

130

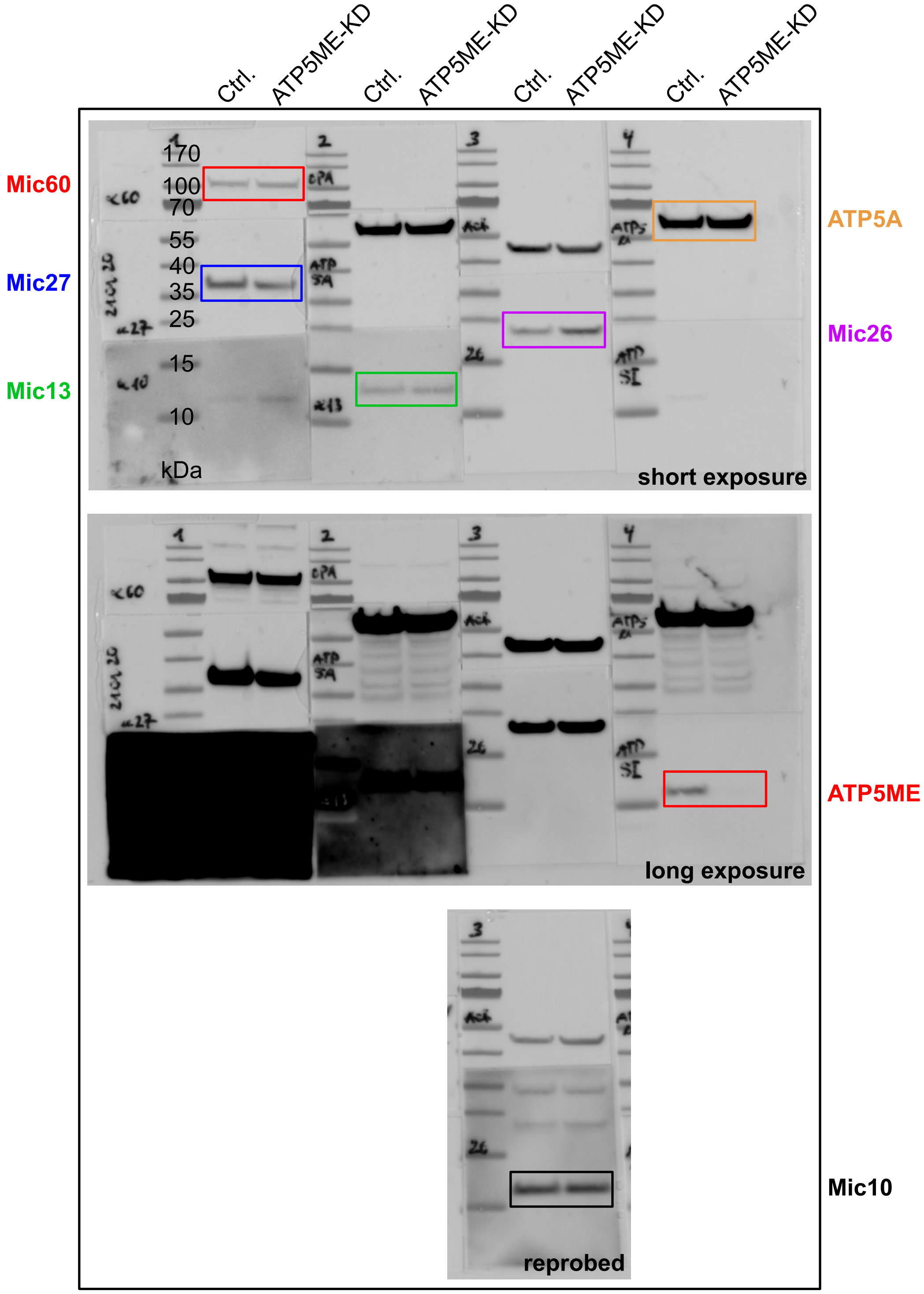

Supplement: Supplementary file 19 — Source Data for Expanded View [file EMBJ-39-e104105-s022.zip › EMBOJ-2019-104105_Source_Data_Figure_EV5.pdf]
